# Recent Updates on Induced Pluripotent Stem Cells in Hematological Disorders

**DOI:** 10.1155/2019/5171032

**Published:** 2019-05-02

**Authors:** Methichit Wattanapanitch

**Affiliations:** Siriraj Center for Regenerative Medicine, Research Department, Faculty of Medicine Siriraj Hospital, Mahidol University, Bangkok 10700, Thailand

## Abstract

Over the past decade, enormous progress has been made in the field of induced pluripotent stem cells (iPSCs). Patients' somatic cells such as skin fibroblasts or blood cells can be used to generate disease-specific pluripotent stem cells, which have unlimited proliferation and can differentiate into all cell types of the body. Human iPSCs offer great promises and opportunities for treatments of degenerative diseases and studying disease pathology and drug screening. So far, many iPSC-derived disease models have led to the discovery of novel pathological mechanisms as well as new drugs in the pipeline that have been tested in the iPSC-derived cells for efficacy and potential toxicities. Furthermore, recent advances in genome editing technology in combination with the iPSC technology have provided a versatile platform for studying stem cell biology and regenerative medicine. In this review, an overview of iPSCs, patient-specific iPSCs for disease modeling and drug screening, applications of iPSCs and genome editing technology in hematological disorders, remaining challenges, and future perspectives of iPSCs in hematological diseases will be discussed.

## 1. Introduction

Pluripotent stem cells (PSCs) including embryonic stem cells (ESCs) and induced pluripotent stem cells (iPSCs) have unlimited self-renewal and proliferation properties as well as an ability to differentiate into mature cell types of all three embryonic germ layers [1, 2]. PSCs offer great potentials to generate clinically relevant number of cells and could provide an alternative source of cells for regenerative medicine [3, 4]. Currently, patient-specific iPSCs can be achieved by reprogramming of adult somatic cells by ectopic expression of pluripotency-associated transcription factors including OCT4, SOX2, KLF4, and c-MYC [2]. The reprogrammed iPSCs have similar characteristics as human ESCs (hESCs) in terms of their self-renewal and differentiation potentials. These patient-specific iPSCs can bypass previous limitations including immunological rejection and ethical barriers that impede the use of hESCs. In addition, they would allow better understanding of mechanisms underlying several human genetic, malignant, and nonmalignant diseases. Recently, genome editing technologies have been applied to correct the mutation of disease-specific iPSCs to create gene-corrected iPSCs, which can be used for autologous cell-based therapy. This review is aimed at providing an update on cellular reprogramming in basic research and potential applications in hematological disorders.

## 2. Generation of Patient-Specific iPSCs

Reprogramming process involves ectopic expression of pluripotency-associated genes including *OCT3/4*, *SOX2*, *KLF4*, and *c-MYC* into somatic cells. Initially, Takahashi and colleagues performed reprogramming in mouse and human fibroblasts using retroviral transduction as a delivery method [2, 5]. One of Yamanaka's factor, c-MYC, is a protooncogene which confers a risk of tumor formation once it gets reactivated. Yu and colleagues reported the use of *NANOG* and *LIN28* to replace *KLF4* and *c-MYC* for reprogramming human fibroblasts, thus providing a safer alternative for clinical applications [6]. The retroviral and lentiviral systems can result in genomic integration of transgenes, therefore increasing the risk of insertional mutagenesis. The lentiviral method has advantages over the retroviral method since it can infect both dividing and nondividing cells giving higher reprogramming efficiency and providing an opportunity for transgene excision via *Cre-loxP* recombination [7, 8]. Previous studies demonstrated that the transcriptomic profiles of human iPSCs generated by nonintegrating methods are more closely similar to those of the hESCs or the fully reprogrammed cells than those of the iPSCs generated from integrating methods [9]. To facilitate future clinical applications, nonintegrating delivery methods such as adenovirus [10, 11], episomal plasmids (Epi) [12], minicircle DNA vectors [13], piggyBac transposons [14], proteins [15], synthetic mRNAs [16, 17], Sendai virus (SeV) [18, 19], and microRNA mimics [20, 21] have been developed. Each reprogramming strategy has its advantages and disadvantages [22, 23]. Factors determining which reprogramming method is suitable to use are the number and type of starting cells, the reprogramming efficiency, footprint, and long-term translational goals [23]. Reprogramming efficiencies of the nonintegrating methods such as adenoviral vectors (0.0002% [10]), minicircle DNA vectors (0.005% [13]), and proteins (0.001% [15]) are very low. It is also labor intensive and technically challenging to synthesize large amounts of proteins for reprogramming. Of these nonintegrating methods, Epi, mRNA, and SeV are more commonly used and were evaluated systematically by Schlaeger et al. [22]. The efficiency of the mRNA-based reprogramming was the highest (2.1%), followed by SeV (0.077%) and Epi (0.013%) as compared to the lentiviral reprogramming (Lenti) (0.27%). However, the mRNA-based method is not so reliable, as the success rate was significantly lower than other methods (mRNA 27%, SeV 94%, Epi 93%, and Lenti 100%). In terms of workload, the SeV method required the least hands-on time until the colonies were ready for picking whereas the mRNA method required the most hands-on time due to the need for daily transfection for 7 days [16, 17]. Importantly, the mRNA method failed to reprogram hematopoietic cells. Therefore, the SeV, Epi, or Lenti reprogramming must be used for specific hematological diseases that require blood cells for reprogramming. For clinical translation, Epi reprogramming is the most cost-effective and well-suited because the process can be made compliant with current good manufacturing practice (cGMP) [22]. Recently, the CTS CytoTune-iPS 2.1 SeV reprogramming suitable for clinical and translational research is commercially available. However, the clinical-grade kit is very expensive; therefore, the method is not widely used in clinical trials. In 2014, the first clinical trial to treat a patient with neovascular age-related macular degeneration (AMD) used autologous iPSCs generated using nonintegrating Epi vectors, which were proven to be safe for the patient [24].

Generation of patient-specific iPSCs requires somatic cells such as fibroblasts [25] or peripheral blood mononuclear cells [26, 27], as starting materials. There have been reports of other somatic cell types used for the derivation of iPSCs including umbilical cord blood [28, 29], bone marrow [30], amniotic fluid or chorionic villus sample-derived cells from prenatal diagnosis [31], stomach and liver cells [32], neural stem cells [33, 34], and endothelial cells [35]. In order to obtain these cells, invasive procedures performed by medical professionals are required. Recently, easily accessible and noninvasive cell sources including keratinocytes from plucked hair [36–38] and exfoliated renal epithelial cells from urine samples [39–41] were isolated for iPSC reprogramming, thus allowing simple and noninvasive sample acquisition. These approaches offer advantages especially when subjects are infants or individuals with bleeding disorders. Reprogramming efficiency of each somatic cell type usually varies depending on the endogenous factors that they express, e.g., neural stem cells which endogenously express Sox2 require only Oct4 and/or Klf4 during the reprogramming process [34, 42]. The differentiation stages of somatic cells also determine the reprogramming efficiency, e.g., hematopoietic stem cells or progenitor cells can be reprogrammed with higher efficiency than terminally differentiated B cells or T cells [43]. Despite various cell types used, fibroblasts and peripheral blood mononuclear cells remain the gold standard because of their ease of isolation and reprogramming. A recent study revealed that aberrant hypermethylation in undifferentiated iPSCs acquired during reprogramming process was found to be a crucial factor that affected hematopoietic differentiation capacity, irrespective of the starting cell type. However, iPSCs derived from blood cells were unlikely to acquire aberrant DNA methylations, and these cells had higher hematopoietic differentiation capacity when compared with iPSCs from other parental tissues. In addition, the reprogramming methods were associated with aberrant DNA methylation and maturation capacity; the Epi and SeV methods gave rise to iPSCs with various aberrant DNA methylation levels and hematopoietic differentiation capacity whereas the retroviral reprogramming gave rise to iPSCs with high aberrant DNA methylation and attenuated differentiation capacity [44]. Therefore, it is crucial to identify the starting cell types and reprogramming methods to generate iPSC lines that are suitable for specific applications.

## 3. iPSCs as Disease Models for Hematological Disorders

Conventionally, transgenic animal models have been used to elucidate disease pathophysiology. However, many of these models do not completely recapitulate disease phenotypes due to fundamental differences between species. In order to study hematological diseases, which affect hematopoietic stem/progenitor cells (HSPCs) in the bone marrow, these cells have to be expanded *ex vivo*. However, during the past two decades, there had been no robust method for maintaining these HSPCs *ex vivo* in their multipotent stage [45, 46]. This becomes an important issue especially for diseases affecting mainly the bone marrow such as idiopathic myelofibrosis or aplastic anemia where the tissue samples are really scarce. Therefore, most studies have relied on the use of peripheral blood cells, which have a limited lifespan in culture, for studying disease pathology. The lack of protocol to maintain and amplify these primary cells also hinders genetic modifications, which are important tools to study candidate gene function [45, 47].

The advent of iPSC technology has transformed the way we study disease mechanisms by providing more opportunities to generate numerous disease models from patients. Disease-specific iPSCs and their derivatives represent an early stage of disease thus providing very useful information for elucidating pathological events during disease initiation and progression otherwise undetectable in primary cells. For generation of blood disease models, selection of starting somatic cells that carry genetic or acquired mutations is essential. For genetic blood disorders such as sickle cell disease, thalassemia, and X-linked chronic granulomatous disease, disease-specific iPSCs can be generated from both skin biopsy (fibroblasts) and blood. However, for acquired blood diseases such as aplastic anemia, leukemia, myelodysplastic syndrome, myeloproliferative neoplasms, and paroxysmal nocturnal hemoglobinuria, where only certain hematopoietic (stem/progenitor) cells are affected, disease-specific iPSCs can be generated from the abnormal or malignant hematopoietic clones. Typically, samples are taken from the bone marrow or peripheral blood mononuclear cells, which are very heterogeneous and contain a mixture of normal cells and premalignant and malignant clones. These clonal subpopulations can vary among samples depending on the disease progression, remission, administered therapies, or *in vitro* culture. Therefore, characterization of cells by next-generation sequencing is necessary to select the suitable starting cells for reprogramming [48]. In contrast, iPSCs derived from fibroblasts of patients with these acquired diseases do not carry the genetic mutations; therefore, they can serve as germ line controls or can be used for production of disease-free HSPCs for autologous transplantation or generation of immune cells for adoptive immunotherapy [45, 49].  Table 1 summarizes the work on disease-specific iPSCs from patients with genetic and acquired diseases for modeling.

## 4. iPSCs for Drug Screening and Toxicity Testing

**Figure 1:**
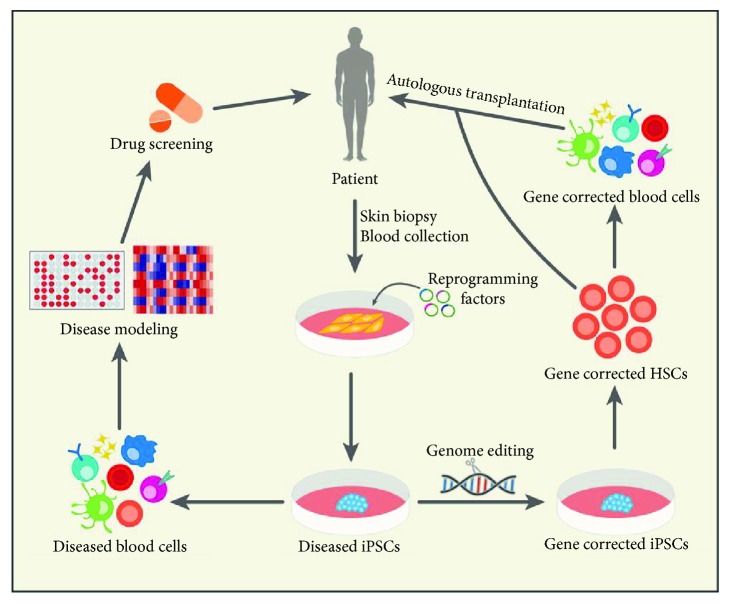
Applications of iPSCs for disease modeling and autologous cell-based therapy. Disease-specific iPSCs can be generated from patients with inherited blood diseases. A panel of disease-specific iPSCs and their derivatives enable high-throughput screening assay against the library of hundreds of thousand compounds. This approach represents a powerful tool for elucidating disease mechanisms and developing new drugs. Alternatively, the genome editing technology can be employed to correct genetic mutations followed by directed differentiation; the gene-corrected iPSC-derived hematopoietic stem cells (HSCs) or other mature blood cells can be transplanted or transfused to the same patient.

A large number of drugs in the market have been developed through cell line-based compound screening and animal testing. However, drug responses tested in animals cannot always be used to predict safety and efficacy in humans. Many drugs failed to enter the market due to unanticipated adverse effects mainly cardiotoxicity and hepatotoxicity in late-stage trials [50]. Advances in iPSC technology allow generation of unlimited supplies of disease-specific iPSCs from heterogeneous backgrounds such as gender and ethnicity. These cells can be differentiated into disease-relevant cell types that demonstrate the disease phenotype similar to primary cells that are hard to access and have limited proliferation. A large panel of disease-specific iPSCs and their derivatives enable high-throughput screening assay against the library of hundreds of thousand compounds. This approach could facilitate the development of novel therapeutics (Figure 1). In addition to efficacy testing in disease-relevant cell types, other cell types such as cardiomyocytes [51, 52] and hepatocytes [53] can be derived from patient's iPSCs. This is very beneficial for evaluating potential drug toxicities at early stages of drug development and could minimize the use of animals during drug testing as well as saving considerable time and costs [54]. These iPSC-based phenotypic assays together with high content screening platform represent a new paradigm for drug discovery. To date, most studies using disease-specific iPSCs for drug screening have been successfully carried out in neuronal diseases such as Alzheimer's disease [55], amyotrophic lateral sclerosis [56], motor neuron disease [57], spinal muscular atrophy [58], familial dysautonomia [59], Rett syndrome [60], and Parkinson's disease [61] as well as in metabolic liver diseases such as hypercholesterolemia [62].

For hematological diseases, JAK kinase inhibitors have been examined in hematopoietic cells differentiated from polycythemia vera- (PV-) derived iPSCs [63]. In this study, peripheral blood mononuclear cells of multiple patients with *JAK2-*V617F mutations were reprogrammed into iPSCs. A panel of iPSCs with different *JAK2* allele compositions including homozygous, heterozygous, and wild type was differentiated into erythroid cells. Samples derived from homozygous and heterozygous *JAK2-*V617F iPSCs underwent enhanced erythropoiesis when compared to the wild-type iPSCs. Once the HSPCs were treated with JAK inhibitors INCB018424 (approved drug), TG101348 (in clinical trial), or CYT387 (in clinical trial), erythroid proliferation was inhibited in a dose-dependent manner. Both INCB018424 and TG101348 were able to block cell proliferation completely at doses ≥ 250 nM whereas CYT387 showed less activity. These data were in accordance with the clinical trial results that the anemia conditions were observed in patients treated with INCB018424 or TG101348. In contrast, the anemia conditions were improved in some myelofibrosis patients treated with CYT387. Furthermore, *JAK2-*V617F iPSC-derived CD34^+^ progenitors were more resistant to JAK inhibitors whereas the derived erythroblasts were sensitive, thus underlying the ineffectiveness of the JAK inhibitors in destroying the diseased clones. More recently, Diamond-Blackfan anemia- (DBA-) iPSCs were generated to model defect in erythropoiesis and screen for novel therapeutics. HSPCs derived from DBA-iPSCs were chemically screened in comparison to the control iPSCs. Treatment with a small molecule inducer of autophagy, SMER28, resulted in enhanced erythropoiesis through the autophagy factor ATG5 and upregulation of globin gene expression in DBA-iPSC-derived erythroid cells [64].

Patient-specific iPSCs were used for modeling myeloid malignancy, which is a disease spectrum ranging from clonal hematopoiesis to myelodysplastic syndrome (MDS) and acute myeloid leukemia (AML). Derivation of iPSCs from the bone marrow or peripheral blood mononuclear cells of patients with different disease stages gave rise to a panel of iPSC lines. A thorough genetic analysis showed that there were normal iPS clones as well as subclones with a variety of genetic mutations and chromosomal abnormalities associated with myeloid neoplasms. Upon hematopoietic differentiation, the high-risk MDS iPSCs had impaired differentiation and reduced clonogenicity affecting erythroid and multilineage progenitors as compared to the low-risk MDS iPSCs, preleukemic or normal iPSCs recapitulating features of disease progression. The disease stage-specific iPSCs were used to study the effects of therapeutic intervention such as the hypomethylating agent, 5-azacytidine (5-AzaC), which is the first-line therapy in MDS, and rigosertib, a small molecule inhibitor of RAS signaling pathways currently in clinical trials for the treatment of high-risk MDS. Treatment with 5-AzaC or rigosertib in HSPCs derived from different disease stage-specific iPSCs resulted in different therapeutic effects [65]. These studies demonstrated the usefulness of disease-specific iPSCs as a powerful tool for elucidating potential drug mechanisms and developing novel therapeutics.

## 5. iPSCs as an Alternative Source for Autologous Cell-Based Therapy

Hematopoietic stem cell transplantation (HSCT) has been used as a standard of care for the treatment of genetic, malignant, and nonmalignant hematological diseases such as multiple myeloma, lymphoma, aplastic anemia, myeloproliferative disorders, myelodysplastic syndromes, thalassemia, Wiskott-Aldrich syndrome (WAS), sickle cell anemia (SCD), severe combined immunodeficiency (SCID), and autoimmune disorders [98]. HSCs are multipotent stem cells, which are able to self-renew and give rise to all the blood progenitors and mature blood cells. HSCs can be directly obtained from the bone marrow of adults, mobilized peripheral blood and cord blood during normal delivery. Since the bone marrow biopsy is invasive, the granulocyte colony-stimulating factor- (GCSF-) mobilized peripheral blood is more commonly used for most autologous and allogeneic transplantation. However, if the suitable donors with HLA matches are not found, HSCs from cryopreserved cord blood can also be used as an alternative source because they are readily available and cord blood transplantation requires less stringent HLA matching than bone marrow or peripheral blood [99, 100]. Nevertheless, the major limitation of cord blood HSCs is the low number of HSCs in the stored units. Therefore, infusion of two partially HLA-matched cord blood units is required for transplantation into an adult patient [101–104].

Despite the success in the HSCT, many patients who received allogeneic HSCT have suffered from major complications such as acute and chronic graft-versus-host diseases (GVHD), which can lead to significant morbidity and mortality [105]. In contrast, autologous HSCT has lower mortality rate as compared to allogeneic HSCT (less than 2% vs. 10%, respectively) [98] and fewer highly morbid immune responses from delayed engraftment. Therefore, autologous HSCs are a good candidate. However, for genetic blood diseases, genetic correction in patient's HSCs is necessary prior to autologous transplantation. At the moment, the main hurdles impeding the wider clinical applications are the challenge of HSPC expansion in culture [46, 105]. These limitations necessitate an unlimited renewable source of surrogate cells for transplantation. iPSCs provide an inexhaustible source of autologous cells that are amenable for genetic correction and can be subsequently directed to differentiate to HSPCs. For certain acquired blood diseases such as paroxysmal nocturnal hemoglobinuria (PNH) [106] or acute myeloid leukemia (AML) [107], iPSCs derived from mutation-free somatic cell sources such as fibroblasts can be used to generate disease-free iPSCs and healthy HSPCs for autologous transplantation. In contrast, iPSCs from genetic blood diseases require gene therapy or correction before differentiation into HSPCs.

In 2007, Hanna and colleagues demonstrated the first proof of principle for the treatment of sickle cell anemia by combining autologous iPSCs from humanized sickle cell anemia mouse model with gene therapy to correct sickle cell mutation in iPSCs. The corrected iPSCs were then differentiated into HSPCs and transplanted into the irradiated mouse with sickle cell anemia to improve all hematological and systemic parameters of sickle cell anemia [108]. Early studies using patient-specific iPSCs as a potential source for autologous cell-based therapy relied on the use of low-efficiency homologous recombination [109] or lentiviral gene therapy [110, 111]. A drawback of the lentiviral gene therapy system is random integration of a functional gene into the genome, which can result in undesired mutations. Over the past few years, the emergence of genome editing technology such as zinc-finger nucleases (ZFNs), transcriptional activator-like effector nucleases (TALENs), or clustered regularly interspaced short palindromic repeat (CRISPR)/Cas9 has opened up the opportunity to correct genetic mutation in iPSCs. This technology relies on artificial endonuclease enzymes that specifically target the DNA sequence and create DNA double-strand breaks (DSBs). The DSBs can then be repaired by an error-prone process called nonhomologous end-joining (NHEJ) in the absence of DNA template, which leads to insertions or deletions (indels). Alternatively, the target sequence can be repaired by introducing a homologous repair template via homology-directed repair (HDR). ZFNs and TALENs are based on DNA-binding proteins and therefore involve protein design and synthesis, which are difficult and labor intensive. On the other hand, CRISPR/Cas9 system, which relies on short guide RNAs (gRNA) to drive RNA-binding Cas9 nuclease to precisely target DSB, has been reported to be much more efficient and easier to design, making rapid adoption by laboratories around the world [112, 113]. Recent studies demonstrated the use of genome editing tools and iPSC technology for targeting monogenic blood diseases.  Table 1 summarizes the studies using disease-specific iPSCs and the genome editing technology to correct genetic mutations of blood diseases followed by directed differentiation of the gene-corrected iPSCs into HSPCs or relevant blood cell types. In most studies, the gene-corrected iPSCs and their derivatives showed restoration of gene and protein expressions. These approaches therefore offer promises for autologous cell-based therapy (Figure 1).

In order to apply iPSCs for blood disease modeling and cell-based therapy, generation of the most desired cell types including HSPCs and their progenies is required. These cells must be efficiently generated and expanded to clinical scale. To date, the biggest challenge that hampers clinical use of iPSC-derived HSPCs is to generate functional HSPCs that are expandable, transplantable, and engraftable. Over the last decade, various hematopoietic induction protocols including stromal cell- (feeder-) based [114, 115], embryoid body- (EB-) based [116–118], and chemically defined protocols [119, 120] have been reported with varying efficiencies. However, these protocols produced short-lived progenitors, which recapitulate primitive hematopoiesis that occurs in the extraembryonic yolk sac. These progenitor cells can only give rise to myeloid cells and nucleated erythrocytes not the lymphoid lineage and lack repopulating and engraftment potentials [121]. Later, stage-specific induction protocols recapitulating hematopoietic ontogeny have been introduced. These protocols rely on the use of cytokines and morphogens such as bone morphogenetic protein 4 (BMP4) to promote mesoderm specification (KDR^+^/CD235a^−^) and the GSK-3*β* inhibitor (CHIR99021, a Wnt agonist) or TGF*β* inhibitor (SB-431542) during the same timeframe to promote definitive hemogenic endothelium (HE, CD34^+^/CD43^−^/CD73^−^/CD184^−^) while inhibiting primitive hematopoiesis. Addition of vascular endothelial growth factor (VEGF), fibroblast growth factor 2 (FGF2), and hematopoietic cytokines further specifies the HE cells toward HSPCs, CD34^+^/CD43^+^, through the process known as endothelial-to-hematopoietic transition (EHT) [122–126]. Most of the stepwise protocols give rise to larger numbers of CD34^+^/CD43^+^ HSPCs as compared to the OP9 coculture system. However, there has been no report on the engraftment potential of iPSC-derived HSPCs from these stepwise differentiation protocols. In contrast, using *in vivo* differentiation approach via teratoma formation, HSPCs with engraftable potential and multilineage reconstitution were generated [127, 128]. However, such process is variable and these cells are not applicable for future clinical setting.

More attempts have been made to identify the combination of transcription factors that can reprogram the somatic cells to HSC-like cells, the so-called induced HSCs (iHSCs). These approaches involve respecification of somatic cells to functional HSPCs. Daley's group respecified iPSC-derived CD34^+^/CD45^+^ myeloid progenitors by ectopic expression of the five transcription factors, *HOXA9*, *ERG*, *RORA*, *SOX4*, and *MYB*, toward multilineage progenitors that can be expanded *in vitro* and engrafted *in vivo*. These five factors promoted only short-term engraftment of erythroid and myeloid cells. The erythroid precursors were matured, underwent enucleation, and expressed adult hemoglobin [129]. In another report, they generated HSPCs from iPSC-derived HE using a combination of seven transcription factors, *ERG*, *HOXA5*, *HOXA9*, *HOXA10*, *LCOR*, *RUNX1*, and *SPI1*. These factors supported multilineage/long-term engraftment and reconstitution of HE undergoing endothelial-to-mesenchymal transition (EHT) upon transplantation into primary and secondary sublethally irradiated NSG mice [130]. More recently, using only a single factor, *MLL-AF4*, iPSC-derived blood cells can be respecified toward long-term engraftable iHSPCs with reconstitution potential toward both myeloid and lymphoid lineages. However, these cells are prone to leukemic transformation during the long-term engraftment period suggesting that the cells are genomically unstable. Interestingly, the genetic aberrations were not found in the *in vitro-*derived iHSPCs [131]. This finding necessitates further investigations into cellular identity of the iHSPCs and underlying mechanism of leukemic transformation upon transplantation. Therefore, more precise knowledge of supportive cues and transcription factors involved during adult-type definitive hematopoiesis is necessary for generation of safe and functional HSPCs from iPSCs [121, 132].

Apart from efforts to generate HSPCs, differentiation protocols to various blood cell types such as red blood cells (RBCs) [133–135], platelets [136–139], T lymphocytes [140–145], and natural killer (NK) cells [146–148] have also been reported. Since RBCs and platelets lack nucleus, they have lower risks of tumorigenesis. Production of universal donor RBCs and platelets generated from iPSCs of blood group O Rh^−^ donors represents an inexhaustible supply for transfusion medicine. However, clinical applications of iPSC-derived RBCs are hindered by terminal maturation of iPSC-derived RBCs, which do not enucleate efficiently and still express mainly embryonic and fetal hemoglobin [134, 135, 149]. Similarly, the major limitation of generation of universal platelets has been inefficient maturation of iPSC-derived megakaryocytes to platelets, which makes the large-scale manufacturing procedure challenging [150]. The forward programming strategy in PSCs by exogenous expression of three transcription factors, *GATA1*, *FLI1*, and *TAL1*, efficiently enhanced production of megakaryocytes allowing the release of functional platelets to large quantities suitable for clinical applications [138]. More recently, turbulence-controllable bioreactors were applied to enhance shedding of platelets and allowed scale up of platelet production to clinically relevant numbers [139]. Generation of cytotoxic T lymphocytes (CTLs) and NK cells from iPSCs has been shown to provide a large supply of rejuvenated cells for adoptive immunotherapy. To date, there have been a number of reports generating iPSC-derived antigen-specific CTLs for the treatment of cancers or infectious diseases including melanoma [144], acute myeloid leukemia [141], hepatocellular carcinoma [145], and EBV [141] and HIV [142] infection. The obtained CTLs had higher proliferation and longer telomere as compared to the original T cells and expressed central memory T cell markers (CCR7, CD27, and CD28), not the exhaustion marker (PD-1) [142]. Recently, chimeric antigen receptor (CAR) technology has been applied for engineering iPSCs to generate CAR-T cells [143] and CAR-NK cells [148] with increased specificity and cytotoxicity for adoptive immunotherapy. Currently, protocols for directed differentiation of iPSCs to functional T or NK cells have relied on the use of mouse stromal cells such as OP9 cells or OP9 cells expressing Notch ligand Delta-like-1 or 4 (OP9-DL1 or DL4) [147, 151, 152]. For clinical application, it is necessary to avoid the use of serum and animal cells for coculturing. To overcome this issue, attempts have been made to replace serum and stromal cells with a fully defined engineered thymus-like niche consisting of recombinant vascular cell adhesion molecule 1 (VCAM-1) and DLL4 for T cell differentiation. This system enabled generation of CD7^+^ progenitor T cells (proT cells) from cord blood CD34^+^ HSPCs [153]. Although the *in vitro* maturation of proT cells to functional T cells has not been demonstrated, this approach provides an important step toward fully defined and xeno-free differentiation platform that can be applied for future therapeutic uses. Similar to *in vitro* generation of HSPCs, the production of functional hematopoietic cells is still a very inefficient process especially in terms of differentiation efficiency and *ex vivo* expansion to clinical scale. To overcome these limitations, more detailed knowledge about embryonic and fetal hematopoiesis during human development is necessary.

## 6. Challenges and Future Perspectives of iPSC Applications

Since the discovery of iPSCs, several progresses have been made to bring iPSCs into clinics. However, there are still important challenges and issues that have to be addressed including the development of safe and clinically relevant iPSCs and generation of functional HSCs and their progenies. For clinical applications, culture and isolation of somatic cells as well as reprogramming process must be xeno-free and clinical grade and performed under good manufacturing practice (GMP) standards. Methods for generation of iPSCs must be integration-free in order to avoid the risks of insertional mutagenesis and transgene reactivation, which can result in tumor formation. In addition, long-term maintenance of iPSC culture can result in the acquisition of chromosomal abnormalities and changes in copy number variants. Standard karyotyping analysis is unable to detect such small chromosomal aberration; therefore, routine examination such as whole genome screening using comparative genomic hybridization is required. After differentiation into specific cell types, DNA methylation and gene/protein expression profiles as well as functional assay of the iPSC-derived cells should be validated and compared with those of the original tissue. Furthermore, tumorigenic potential of residual pluripotent cells in the differentiated cells should be validated in animals before transplantation into the patient [24, 154]. Elimination of these unwanted pluripotent cells is crucial prior to use in the clinical setting. Approaches including positive selection of differentiated cells using specific surface markers [155], selective elimination of residual undifferentiated cells using compounds [156–159] or selective media [160, 161], and engineered safety switches such as inducible suicide genes in undifferentiated cells [162, 163], suicide-inducing virus-like particles [164], lectin-toxin fusion protein [165], or microRNA-302 switch [166] could be performed to minimize the risk of tumor formation. More detailed approaches have been extensively reviewed in Jeong et al. [167].

Application of iPSCs in autologous cell-based therapy represents an ideal approach for regenerative medicine since patients do not require long-term immunosuppressive drugs. The first clinical trial using autologous iPSC-derived retinal pigmented epithelial cells for the treatment of macular degeneration has proven to be safe. The patient has no adverse effect after the treatment. However, for more common diseases, autologous therapy may not be practical due to the high cost and the long period of time spent in the manufacturing process: generation, characterization, differentiation into relevant cell types, scale up, and careful validation. Recently, advances in iPSC therapy are moving toward allogeneic approaches in order to bring down the manufacturing cost and reduce the production time. Broad applicability of iPSCs can be achieved by establishment of clinical grade iPSC banking from selected HLA homozygous donors with blood group O to cover the majority of potential recipients [168, 169]. Practically, this approach will be very challenging and requires extensive efforts to establish such iPSC bank especially in populations with more diverse genetic backgrounds. Therefore, the most viable approach is to have a universal iPSC line, which is prepared in advance and can be given to patients on demand regardless of their HLA haplotypes. Several groups generated HLA-engineered stem cells that are invisible to both humoral and cellular alloimmune responses by employing a short-hairpin mRNA (shRNA) to knockdown [137, 170] or genome editing technology to knockout the *β*2-microglobulin (*B2M*) gene [136, 171, 172], which is responsible for the HLA class I light chain as well as the *CIITA* gene, which is a master regulator of HLA class II molecule [173, 174]. These HLA-edited cells are susceptible to lysis by recipient's NK cells due to missing self-response. Forced expression of less polymorphic HLA-E molecule in HLA-engineered iPSCs has been shown to prevent NK cell lysis [175]. More recently, ectopic expression of CD47 in mouse and human B2M^−/−^ and CIITA^−/−^ iPSCs rendered the cells hypoimmunogenic to T cells and all innate immune responses. Upon transplantation of the engineered hiPSCs and their differentiated derivatives into allogeneic humanized mouse model, the recipients did not elicit any cellular or humoral immune response. The grafts showed long-term survival (50 days) [173]. However, the complete escape of immune surveillance raises some safety concerns regarding the risks of tumor formation and viral infection [172, 173]. Strategy such as targeted disruption of *HLA-A/B* genes and retaining *HLA-C* gene in iPSCs can suppress T and NK cell activity while preserving antigen presentation to a certain extent [176]. Alternatively, inducible kill switches can be incorporated into the HLA-engineered cells. Altogether, further refinement of these approaches will increase donor compatibility, reduce the use of immunosuppressive drugs, and ultimately provide a universal source of cells for regenerative medicine.

## 7. Conclusions

The iPSC technology provides PSCs that can be differentiated to any mature cell types, which are genetically and phenotypically identical to the patients. Generation of hematological disease-specific iPSCs helps increase our understanding of disease mechanism and progression. Together with recent advances in high-throughput screening and genome editing technologies, these patient-specific iPSCs provide a powerful tool to complement *in vivo* animal models for drug screening, toxicity testing, and development of personalized medicine. Although there are challenges regarding the efficiency of generation of HSPCs and their mature functional blood cells, scale-up process, and validation of clinical-grade cells as well as the concern about immunogenicity to overcome, iPSCs still serve as an ideal source and offer great opportunities for future regenerative medicine.

## Figures and Tables

**Table 1:** iPSCs as disease models and applications of gene therapy or genome editing for hematological disorders.

Disorders	Affected gene(s)	Phenotype assessment	Gene therapy/correction	Ref
AML	*MLL*	AML-iPSCs lacked leukemic potential but reacquired the ability upon hematopoietic differentiation *in vivo*.	N/A	[66]

CGD	*CYBB*	CGD iPSC-derived neutrophils lacked ROS production.	ZFN-mediated *CYBB* gene correction substantially restored neutrophil ROS production.	[67]

CML	*BCR-ABL*	CML-iPSCs and hematopoietic cells were used as models for studying mechanism leading to leukemic stem cell survival in the presence of tyrosine kinase inhibitor.	N/A	[68–71]

DBA	*RPS19* and *RPL5*	Mutant iPSCs exhibited defects in ribosomal subunit assembly and impaired erythropoiesis upon differentiation.	ZFN-mediated *RPS19* and *RPL5* gene correction alleviated abnormalities in ribosome biogenesis and hematopoiesis.	[72]
	*RPS19* and *RPL5*	DBA-iPSCs showed altered TGF*β* signaling, aberrant ribosome biogenesis, and impaired erythropoiesis when compared to the wild-type iPSCs.	Ectopic expression of both genes in the “safe harbor” AAVS1 site restored the level of SMAD4, which is the major effector of the canonical TGF*β* signaling pathway.	[73]

FPD/AML	*RUNX1*	FPD-iPSCs are uniformly defective in hematopoietic progenitor (HP) emergence and megakaryocyte (MgK) differentiation.	Overexpression of *RUNX1* rescued emergence of HP cells but partially restored MgK maturation.	[74]

HA	*F8*	Endothelial cells (ECs) derived from HA-iPSCs lacked *F8* transcript and FVIII protein.	Targeted chromosomal inversions restored *F8* transcript and FVIII protein secretion in the corrected iPSC-derived ECs.	[75–78]
	*F8*	Endothelial cells (ECs) derived from HA-iPSCs had undetectable levels of FVIII gene expression and secretory protein.	Lentiviral gene therapy in HA-iPSCs restored FVIII secretion in the corrected iPSC-derived ECs both *in vitro* and *in vivo* in immune-deficient HA mouse model.	[79]

HB	*FIX (F9)*	Hepatocyte-like cells derived from HB-iPSCs could not secrete clotting factor IX.	CRISPR/Cas9-based point correction or knock-in full-length FIX cDNA in HB-iPSCs restored clotting factor IX secretion. Upon transplantation, human albumin and factor IX were detected up to 9-12 months in a mouse model of HB.	[80]
	*FIX (F9)*	Hepatocyte-like cells derived from HB-iPSCs could not secrete clotting factor IX.	CRISPR/Cas9-mediated correction of *FIX* point mutation or targeted knock-in full-length FIX cDNA at AAVS1 locus in HB-iPSCs restored clotting factor IX secretion in the corrected iPSC-derived hepatocyte-like cells.	[81, 82]

MDS	Loss of chromosome 7q (del(7q))	MDS-iPSCs had impaired hematopoietic differentiation potential and clonogenic capacity and increased cell death upon differentiation.	Spontaneous acquisition of an extra chromosome 7 fully restored hematopoietic differentiation potential of the MDS-iPSCs.	[65, 83]

PNH	*PIGA*	*PIGA*-iPSCs were unable to produce hematopoietic cells or mesodermal cells expressing KDR/VEGFR2 and CD56 markers.	N/A	[84]

PV	*JAK2 (V617F)*	iPSC-derived hematopoietic cells exhibited enhanced erythropoiesis.	N/A	[63, 85, 86]

SCD	*HBB*	N/A	Correction of sickle point mutation by CRISPR/Cas9 or TALENs allowed HBB protein production in the corrected iPSC-derived erythrocytes.	[87, 88]
SCID-X1	*JAK3*	*JAK3*-deficient iPSCs had a complete block in early T cell development.	Correction of *JAK3* gene by CRISPR/Cas9 restored normal T cell development.	[89]
	*IL-2Rγ*	*IL-2Rγ* mutant iPSCs could not differentiate to functional lymphocytes.	TALEN-mediated *IL-2Rγ* gene correction restored the production of mature NK cells and T cell precursors.	[90]

Thalassemia	*HBB*	Erythrocytes differentiated from homozygous beta thalassemia-iPSCs lacked *HBB* gene and protein expressions.	Correction of *HBB* mutation by CRISPR/Cas9 restored HBB gene and protein expression in the corrected iPSC-derived erythrocytes.	[91–93]
	*HBB*	Double heterozygous *HbE*/*β*-thalassemia iPSCs produced lower hematopoietic progenitor and erythroid cells than the wild-type iPSCs under feeder-free HSPC differentiation system.	Correction of *HBE* mutation by CRISPR/Cas9 restored the number of hematopoietic progenitor and erythroid cells.	[94]
	*HBA*	Homozygous alpha thalassemia iPSC-derived erythroid cells expressed no *α*-globin chains.	ZFN-mediated *HBA* gene correction resulted in restoration of globin chain imbalance in the corrected iPSC-derived erythroid cells.	[95]

WAS	*WAS*	WAS-iPSCs exhibited defects in platelet production.	Lentiviral gene therapy in WAS-iPSCs improved structures of proplatelet and increased the platelet size.	[96]
	*WAS*	WAS-iPSCs exhibited deficient T lymphopoiesis and natural killer (NK) cell differentiation and function.	ZFN-mediated *WAS* gene correction restored T and NK cell differentiation and function.	[97]

AML: acute myeloid leukemia; CGD: chronic granulomatous disease; CML: chronic myeloid leukemia; DBA: Diamond-Blackfan anemia; FPD/AML: familial platelet disorder/acute myeloid leukemia; HA: hemophilia A; HB: hemophilia B; MDS: myelodysplastic syndromes; PNH: paroxysmal nocturnal hemoglobinuria; PV: polycythemia vera; SCD: sickle cell disease; SCID: severe combined immunodeficiency; WAS: Wiskott-Aldrich syndrome.
